# Comprehensive Transcriptome Profiling Uncovers Molecular Mechanisms and Potential Candidate Genes Associated with Heat Stress Response in Chickpea

**DOI:** 10.3390/ijms24021369

**Published:** 2023-01-10

**Authors:** Himabindu Kudapa, Rutwik Barmukh, Vanika Garg, Annapurna Chitikineni, Srinivasan Samineni, Gaurav Agarwal, Rajeev K. Varshney

**Affiliations:** 1 Center of Excellence in Genomics & Systems Biology, International Crops Research Institute for the Semi-Arid Tropics (ICRISAT), Patancheru 502324, India; 2 Murdoch’s Centre for Crop & Food Innovation, State Agricultural Biotechnology Centre, Food Futures Institute, Murdoch University, Murdoch, WA 6150, Australia; 3 Plant Biology Laboratories, College of Natural Science, Michigan State University, East Lansing, MI 48824, USA

**Keywords:** legumes, *Cicer arietinum*, candidate genes, differentially expressed genes (DEGs), heat stress, molecular mechanisms, RNA-seq, signaling pathways

## Abstract

Chickpea (*Cicer arietinum* L.) production is highly susceptible to heat stress (day/night temperatures above 32/20 °C). Identifying the molecular mechanisms and potential candidate genes underlying heat stress response is important for increasing chickpea productivity. Here, we used an RNA-seq approach to investigate the transcriptome dynamics of 48 samples which include the leaf and root tissues of six contrasting heat stress responsive chickpea genotypes at the vegetative and reproductive stages of plant development. A total of 14,544 unique, differentially expressed genes (DEGs) were identified across different combinations studied. These DEGs were mainly involved in metabolic processes, cell wall remodeling, calcium signaling, and photosynthesis. Pathway analysis revealed the enrichment of metabolic pathways, biosynthesis of secondary metabolites, and plant hormone signal transduction, under heat stress conditions. Furthermore, heat-responsive genes encoding bHLH, ERF, WRKY, and MYB transcription factors were differentially regulated in response to heat stress, and candidate genes underlying the quantitative trait loci (QTLs) for heat tolerance component traits, which showed differential gene expression across tolerant and sensitive genotypes, were identified. Our study provides an important resource for dissecting the role of candidate genes associated with heat stress response and also paves the way for developing climate-resilient chickpea varieties for the future.

## 1. Introduction

Grain legumes are mostly grown in marginal environments and are the major source of nutrition and protein to the human population in several countries of Asia and Sub-Saharan Africa. Chickpea (*Cicer arietinum* L.) is an important food legume crop grown worldwide, particularly in the semi-arid tropics. Globally, chickpea is cultivated on an area of ~14.84 million hectares with a production quantity of ~15.08 million metric tonnes annually [1]. Being a leguminous crop, chickpea provides a rich source of nitrogen, thereby enhancing soil fertility. This crop, however, has low productivity, mainly due to its exposure to a range of abiotic (heat, drought, salinity, etc.) and biotic (Ascochyta blight, Fusarium wilt, pod borer, etc.) stresses [2,3,4,5]. 

Changes in climatic conditions, particularly high temperatures, over the past two decades have tremendously influenced the production and productivity of chickpea [6]. Heat stress during the reproductive stage is a major factor limiting chickpea yields [7]. Flowering and seed setting are the most critical components that are affected by heat stress in chickpea. For instance, the seed yield of chickpea was drastically reduced when the plants were exposed to high temperatures (35 °C) at the flowering and pod development stages [8]. Heat stress also adversely affects pollen viability, fertilization, and seed development leading to a reduction in harvest index. In addition, high temperatures cause partial to complete male reproductive tissue sterility, subsequently causing significant losses of grain yield in crops [9,10]. The development of genetic resources for molecular breeding of heat tolerance is important for crop improvement programs. In the case of chickpea, field screening of reference collection (280 diverse germplasm lines) and early maturing germplasm lines (35) led to the identification of potential heat-tolerant and heat-sensitive lines based on a heat tolerance index (calculated through a multiple regression approach wherein seed yield under heat stress was considered as a function of yield potential and days to 50% flowering) [11,12]. Some promising heat-tolerant lines (e.g., ICC 15614) are being used as donors in chickpea breeding programs for the development of heat-tolerant varieties [13]. However, the genotypic diversity of chickpea global germplasm collections grown under different production environments, particularly those effected by heat/high temperature, have not been completely studied. Importantly, it was reported that the heat tolerance of chickpea is likely to be multi-genic, and the components of heat tolerance are probably controlled by different sets of genes [12]. Hence, the development and characterization of large-scale genomic resources are essential for understanding the genetics of heat tolerance in chickpea. 

Sequencing of *desi* and *kabuli* chickpea genomes has been completed [14,15], and an integrated genome-wide physical, genetic, and genome map has been developed [16]. These resources help with locating the genes responsible for variations in quantitative traits of interest. In addition, during the last decade, large-scale genomic resources including whole-genome sequencing data [17,18], transcriptome assemblies [19,20,21,22], high density genetic maps [23,24], and bin maps [25] have been developed. Further, a comprehensive *Cicer arietinum* gene expression atlas (CaGEA) was developed from 27 tissues covering five major developmental stages of the plant [26]. Such large-scale functional genomic resources can be used to uncover the genetic architecture of complex traits and to identify candidate genes associated with biotic/abiotic stress tolerance for use in crop improvement programs. The majority of the studies on chickpea have focused on genetic dissection of resistance against biotic stresses like Fusarium wilt [27], Ascochyta blight [27,28], and *Helicoverpa armigera* [29]. However, limited studies have been conducted to understand the genetics of abiotic stresses like heat tolerance [7], drought tolerance [3,4,5], and salt tolerance [30,31]. Despite the availability of large-scale genomic resources, there have been finite efforts to understand the genetics of complex traits such as heat stress response. In this regard, the development of genomic resources and their comprehensive analysis are a pre-requisite to facilitate chickpea breeding for enhanced heat tolerance. 

In the present study, we conducted RNA-sequencing (RNA-Seq) of six contrasting heat-responsive chickpea genotypes under control and heat stress conditions at the vegetative and reproductive stages of plant development. Multiple DEGs displaying the development stage- and/or genotype-specific responses to heat stress were identified. Gene ontology (GO) enrichment analysis and pathway analysis uncovered key biological processes and metabolic pathways associated with heat tolerance in chickpea. The overlap of previously identified QTLs with differential gene expression patterns from the current study revealed heat stress-responsive candidate genes. Taken together, this study provides crucial insights into the molecular mechanisms and candidate genes underpinning heat stress response in chickpea.

## 2. Results

### 2.1. Generation, Mapping, and Assessment of RNA-Seq Reads

**Figure 1:**
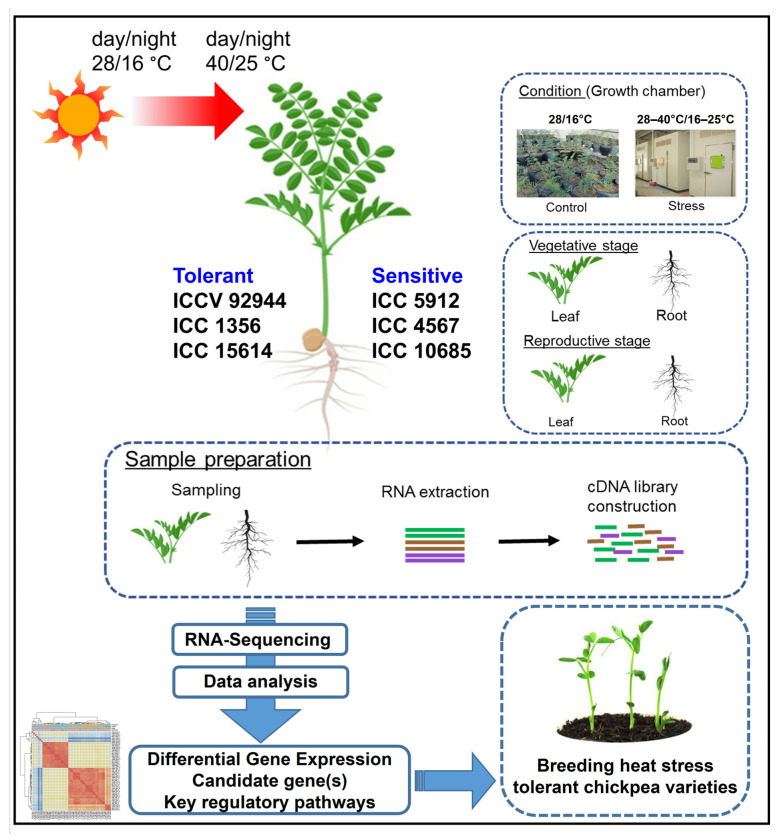
Graphical representation of the experimental design and transcriptome analysis for identifying key genes and molecular mechanisms associated with heat tolerance in chickpea. This figure provides an overview of the experimental conditions, samples/developmental stages targeted, RNA-seq analysis performed, results obtained, and future prospects of the present study.

In this study, paired-end RNA-seq reads were generated from 48 samples (leaf and root) collected at the vegetative and reproductive stages from heat-tolerant (ICCV 92944, ICC 1356, and ICC 15614) and -sensitive (ICC 5912, ICC 4567, and ICC 10685) chickpea genotypes under control and heat stress conditions (Figure 1). A total of 458.85 million reads were generated from the paired-end sequencing of 48 samples (Table 1). About 98.45% (451.70 million) high-quality reads were obtained after applying stringent quality filters, and these reads were used for downstream analysis. All 451.70 million filtered reads were aligned to the chickpea reference genome [14], which resulted in the mapping of 97.41% (440.51 million) of reads (Table 1). These mapping statistics suggested the presence of a high-quality transcriptome sequencing. The sample-wise details of the generated sequence data, filtered reads, reads mapped on the genome, and percent alignment are provided in Table 1. The samples hereafter have been designated as genotype_stage + tissue_condition. For example, the sample 92944_BFL_C denotes genotype ICCV 92944 at the vegetative stage [i.e., before flowering (BF)] with leaf (L) sample collected under control (C) conditions; whereas 92944_AFL_S denotes genotype ICCV 92944 at the reproductive stage [i.e., after flowering (AF)] with leaf (L) sample collected under heat stress (S) conditions.

**Figure 2:**
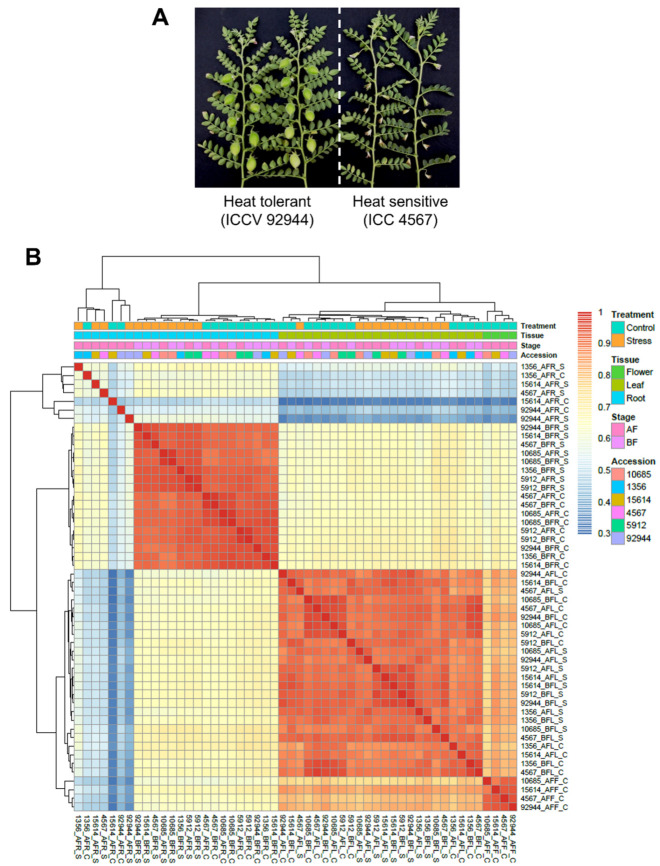
Global transcriptome analysis of 48 samples from six contrasting heat stress-responsive chickpea genotypes. (**A**) Plant phenotype of chickpea heat-tolerant (ICCV 92944) and -sensitive (ICC 4567) genotypes in response to heat stress. (**B**) Heatmap for the transcriptome profiling has been shown based on the hierarchical clustering of Pearson’s correlations (*R*) for the 48 samples. The color scale indicates the degree of correlation. Samples were clustered based on their pairwise correlations. Genes with a normalized expression level FPKM ≥ 1 in at least one of the 48 samples analyzed were designated as expressed and shown in the figure.

The reads (440.51 million) mapped on the chickpea genome were used to generate reference-guided assembly and to analyze global and differential gene expression profiles. The reference-guided assembly generated 31,707 genes, and its comparison with the chickpea genome led to the identification of 28,769 annotated and 2938 novel genes. The normalized expression level, fragments per kilobase of exon per million mapped (FPKM) reads of each gene, was estimated in all 48 samples studied. To exclude genes with low confidence expression values, only those genes with an FPKM ≥ 1 in at least one of the samples analyzed were designated as expressed (Supplementary Table S1). Further, hierarchical clustering of Pearson’s correlation in the transcriptome data was performed for the 48 samples studied (Figure 2). Here, leaf samples (before and after flowering) of all six genotypes under control and heat stress conditions were clustered together, whereas root samples (after flowering) of three tolerant genotypes under control and heat stress conditions, and ICC 4567 under heat stress conditions formed a separate cluster. 

### 2.2. Distribution of DEGs across Six Chickpea Genotypes Contrasting for Heat Stress Response

In order to capture all possible DEGs, we performed differential expression analysis using 104 different pairwise combinations. Of the total number of DEGs obtained, 14,544 were unique and displayed significant differential expression across 104 pairwise combinations. The number of DEGs ranged from 18 (i.e., 7 up-regulated and 11 down-regulated) between 10685_AFR_S and 10685_BFR_S, to 2122 (i.e., 135 up-regulated and 1987 down-regulated) between 15614_AFR_S and 5912_AFR_S (Supplementary Table S2). A substantial variation was detected in the number of genes exhibiting differential expression in leaf and root samples in response to heat stress. 

**Figure 3:**
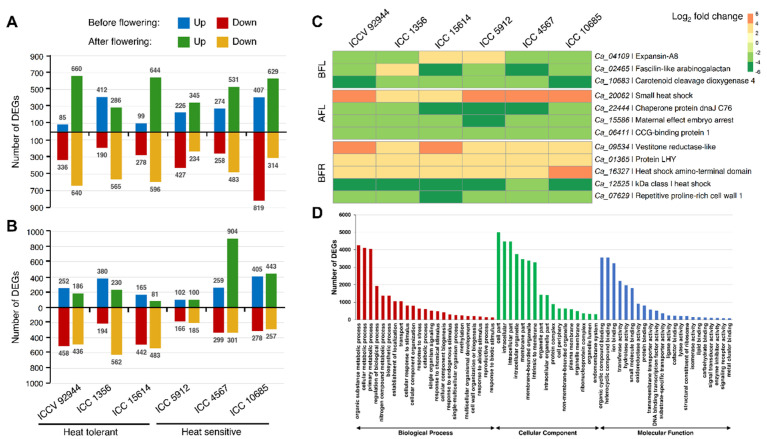
Differentially expressed genes (DEGs) identified in the leaf and root samples of six chickpea genotypes and their functional classification. (**A**) Number of DEGs obtained in the leaves of six chickpea genotypes in response to heat stress at the vegetative and reproductive stages of plant development. The bars above and below the X-axis indicate the number of up-regulated and down-regulated genes, respectively. (**B**) Number of DEGs obtained in the roots of six chickpea genotypes in response to heat stress at the vegetative and reproductive stages of plant development. (**C**) Heat map of DEGs significantly regulated in all six chickpea genotypes under heat stress conditions. The color scale represents log_2_-fold change. BFL, before-flowering leaf; AFL, after-flowering leaf; BFR, before-flowering root. (**D**) Gene ontology (GO) annotation and enrichment analysis of the DEGs. The horizontal axis depicts biological process, cellular component, and molecular function, while the vertical axis represents the number of DEGs associated with each GO term.

In order to identify the distribution of DEGs across six chickpea genotypes in response to heat stress, the gene expression pattern in heat-stressed and control samples was analyzed genotype-by-genotype and independently. In leaf samples before flowering, a total of 421, 602, and 377 DEGs were identified in the tolerant genotypes ICCV 92944, ICC 1356, and ICC 15614, respectively; whereas 653, 532, and 1226 DEGs were observed in the sensitive genotypes ICC 5912, ICC 4567, and ICC 10685, respectively (Figure 3A). In leaf samples after flowering, ICCV 92944, ICC 1356, and ICC 15614 possessed 1300, 851, and 1240 DEGs, respectively; while ICC 5912, ICC 4567, and ICC 10685 had 579, 1014, and 943 DEGs, respectively (Figure 3A). Furthermore, in root samples before flowering, the tolerant genotypes ICCV 92944, ICC 1356, and ICC 15614 showed 710, 574, and 607 DEGs, respectively; while the sensitive genotypes ICC 5912, ICC 4567, and ICC 10685 displayed 268, 558, and 683 DEGs, respectively (Figure 3B). Root samples collected after flowering showed the presence of 622, 792, and 564 DEGs in ICCV 92944, ICC 1356, and ICC 15614, respectively; whereas, 285, 1205, and 700 DEGs were detected in ICC 5912, ICC 4567, and ICC 10685, respectively (Figure 3B). Collectively, these results suggest genotype- and stage-specific responses to heat stress in chickpea.

Based on the results obtained using pairwise analyses of DEGs, the sets of genes of interest were prioritized based on the following criteria: (i) genes that displayed fundamental heat stress responses regardless of the genotype; and (ii) genes that showed differential expression in three tolerant genotypes but responded poorly in three sensitive genotypes. 

### 2.3. Genes Consistently Displaying Significant Differential Expression across all Six Chickpea Genotypes

Among the total set of 3811 heat stress-responsive DEGs detected across six genotypes in the leaf samples before flowering, three genes were consistently and significantly regulated in all six genotypes (Figure 3C). These included genes encoding fasciclin-like arabinogalactan (*Ca_02465*), which are known to play roles in plant growth and development, defense against abiotic stresses, and cell wall biosynthesis [32]; the expansin-A8 protein (*Ca_04109*) that improves cell membrane stability and lowers membrane lipid peroxidation under heat stress [33]; and those encoding carotenoid cleavage dioxygenase 4 (*Ca_10683, Ca_10684*), which produces a photoprotective pigment called apocarotenoid [34]. Further, four genes were conservatively regulated in leaf samples after flowering across all six genotypes, out of 5927 heat-responsive DEGs (Figure 3C). These comprised of genes coding for localized small heat shock protein (*Ca_20062*) and chaperone protein dnaJ C76 (*Ca_22444*), which act as molecular chaperones to promote thermotolerance [35,36]; maternal effect embryo arrest protein (*Ca_15586*), which regulates cell proliferation and controls organ size [37]; and those coding for the CCG-binding protein 1 (*Ca_06411*). Out of the overall set of 3400 DEGs identified across six genotypes in the root samples before flowering, five genes showed consistent significant differential regulation in all genotypes (Figure 3C). This included genes encoding vestitone reductase-like protein (*Ca_09534*), which are involved in the biosynthesis of isoflavonoid phytoalexins [38]; transcription factor LHY (*Ca_01365*), which modulates the circadian period [39]; repetitive proline-rich cell wall 1 (*Ca_07629*), which is involved in mediating cross-connections between cell wall components and in facilitating cell elongation [40]; and those encoding heat shock proteins (*Ca_16327* and *Ca_12525*). No DEGs were found to be conservatively regulated across all six genotypes in root samples after flowering. 

Even though some of these genes were previously shown to be involved in fundamental heat stress response mechanisms [33,35,36,39], they were found to be consistently regulated across all six chickpea genotypes with a varying response to heat stress. Therefore, on the basis of these results, we predict that higher induction or repression of these genes may influence the heat tolerance of chickpea genotypes. 

### 2.4. Genes That Show Significant Regulation in Tolerant Genotypes but Are Not Substantially Regulated in the Sensitive Genotypes

Genes that are responsive to heat stress in the three tolerant genotypes, but not in the sensitive genotypes, represent potential candidates for use in molecular breeding programs. To this end, two genes were found to be significantly regulated in the leaf samples (before flowering) of three tolerant genotypes but were not significantly regulated in the sensitive genotypes (Table 2). These included genes related to pathogenesis-related protein (*Ca_14776*) and adenylate isopentenyl transferase (*Ca_07845*). Further, 14 genes were detected that were significantly regulated in the leaf samples (after flowering) of tolerant genotypes but not significantly so in the sensitive genotypes (Table 2). These included genes involved in metabolic processes, including CTP synthase-like (*Ca_27831*) and serine carboxypeptidase-like (*Ca_00464*); genes encoding transcription factors, NAC family transcription factor 4 (*Ca_06899*), transcription factor TGA4-like (*Ca_09600*), and nuclear transcription factor Y (*Ca_11344*); genes encoding abscisic acid (ABA)-responsive protein ABR18-like (*Ca_02987*); as well as those encoding glycine-rich cell wall structural 1-like protein (*Ca_25834*). In root samples (before flowering) of tolerant genotypes, 10 genes were identified to be significantly regulated, but these genes did not show significant regulation in sensitive genotypes (Table 2). A strong up-regulation of genes encoding cysteine-rich repeat secretory protein 38 (*Ca_14147*) and peroxidase 5-like (*Ca_03546*) was observed, while genes encoding E3 ubiquitin-ligase (*Ca_14738*), dirigent protein 9-like (*Ca_18792*), and pectate lyase 1 (*Ca_16612*) were found to be down-regulated. In addition, cell wall modification-related genes, including arabinogalactan peptide 21-like (*Ca_19160*) and pectinesterase/pectinesterase inhibitor 25 (*Ca_23747*), were found to be significantly regulated in the tolerant genotypes. Furthermore, five genes were found to be significantly regulated in the roots (after flowering) of tolerant genotypes but were not substantially regulated in the sensitive genotypes (Table 2). These included genes coding for ras-related protein RABE1c-like (*Ca_11978*), OBERON-like protein (*Ca_09783*), and glutamine-tRNA ligase (*Ca_05504*), among others.

In addition to the identification of heat-responsive genes that are common across all tolerant genotypes, we detected DEGs that are significantly regulated in a particular tolerant genotype but not in the sensitive genotypes under heat stress conditions. These genes may provide insights into the molecular mechanisms adopted by each tolerant genotype in order to adapt to heat stress. A total of 1616 genes were identified to be significantly regulated in ICCV 92944, but not so in the three sensitive genotypes (Supplementary Table S3). Of these, 816 genes were regulated in the leaves (i.e., 156 genes before flowering and 660 genes after flowering), while 800 genes were regulated in the roots (i.e., 312 genes before flowering and 488 genes after flowering). We identified a strong down-regulation of several cell wall modification-related genes, including L-ascorbate oxidase (*Ca_11122*), pectate lyase (*Ca_24290*), pectinesterase inhibitor domain-containing protein (*Ca_04369*), proline-rich extensin-like protein (*Ca_22519*); and genes involved in the calcium signaling process, including calcium permeable stress-gated cation channel 1-like (*Ca_03393*), calcium-binding protein (*Ca_16032* and *Ca_27828*), and calcium uniporter protein (*Ca_14695*). A strong up-regulation of genes encoding lipolytic enzyme (GDSL esterase/lipase, *Ca_00049*), Ca^2+^ sensor (calmodulin-like protein, *Ca_08153*), and antioxidant enzyme (peroxidase, *Ca_07982*), among others, was observed. A total of 1541 genes were significantly regulated in ICC 1356 but not regulated in the sensitive genotypes (Supplementary Table S4). These included 662 genes in the leaves (i.e., 258 genes before flowering and 404 genes after flowering) and 879 genes in the roots (i.e., 244 genes before flowering and 635 genes after flowering). Here, genes coding for thaumatin-like pathogenesis-related protein 4 (*Ca_05720*), ABA-responsive protein ABR18-like (*Ca_02987*), asparagine synthetase (*Ca_09030*), isoflavone 3’-hydroxylase-like (*Ca_17387*), etc., were significantly down-regulated, whereas those coding for heat stress transcription factor B-2a (*Ca_19774*), aldehyde oxidase (*Ca_05953*), and chaperone protein dnaJ 20 (*Ca_05235*), among others, were highly up-regulated. 

A total of 1675 genes were significantly regulated in ICC 15614, but not in the three sensitive genotypes (Supplementary Table S5). Of the 1675 genes, 930 genes were regulated in leaves (i.e., 182 genes before flowering and 748 genes after flowering) and 745 genes were regulated in roots (i.e., 297 genes before flowering and 448 genes after flowering). The down-regulated genes encoded enzymes involved in sucrose metabolism (acid beta-fructofuranosidase, *Ca_02363*), cell wall remodeling (glucan endo-1,3-beta-glucosidase-like, *Ca_11141*; pectinesterase-like, *Ca_16606*), antioxidants (L-ascorbate oxidase, *Ca_06561*; peroxidase 3-like, *Ca_23217*), and calcium signaling (calcium-binding protein PBP1-like, *Ca_14130*; calcium-dependent protein kinase 28, *Ca_01968*), among others. By contrast, genes encoding for ferric reduction oxidase 2 (*Ca_13723*), cyclin-dependent protein kinase inhibitor SMR11 (*Ca_11286*), ethylene-responsive transcription factor (*Ca_00326*, *Ca_01683*, and *Ca_15022*), etc., were up-regulated. In general, a large number of genes (~70% of total) were found to be significantly regulated in leaves and roots (after flowering) of all tolerant genotypes but were not substantially regulated in the sensitive genotypes. These results imply that the tolerant genotypes undergo a major transcriptional reprogramming during the reproductive stage to improve heat stress tolerance. 

### 2.5. Functional Categorization of DEGs 

The identified DEGs were further functionally classified via gene ontology (GO) and KEGG pathway analysis. The GO enrichment analysis was performed for the 14,544 heat stress-responsive genes. Of the 14,544 significant DEGs obtained, a total of 11,349 genes were annotated to the GO database under the following categories: (i) biological process, (ii) molecular function, and (iii) cellular component. About 4185 GO terms were assigned to 11,349 genes, which were distributed across biological process (8094), molecular function (8931), and cellular component (7593) (Figure 3D). The biological processes, including metabolic process (GO:0008152), cellular process (GO:0009987), response to stimulus (GO:0050896), biological regulation (GO:0065007), localization (GO:0051179), establishment of localization (GO:0051234), cellular component organization or biogenesis (GO:0071840), and developmental process (GO:0032502), among others, were significantly enriched under heat stress. Furthermore, the GO terms catalytic activity (GO:0003824), binding (GO:0005488), DNA binding transcription factor activity (GO:0003700), transporter activity (GO:0005215), structural molecule activity (GO:0005198), enzyme regulator activity (GO:0030234), electron transfer activity (GO:0009055), peroxidase activity (GO:0004601), etc., were significantly enriched under the molecular function category, and the GO terms cell part (GO:0033643), organelle (GO:0043226), membrane (GO:0016020), and extracellular region (GO:0005576), among others, were substantially enriched in cellular component category. 

In order to detect molecular pathways and biological processes regulated under heat stress in chickpea, a pathway analysis of the DEGs was conducted using Kyoto Encyclopedia of Genes and Genomes (KEGG) software [41]. A total of 134 pathways were represented by the DEGs (Supplementary Table S6). Under heat stress conditions, pathway analysis revealed the enrichment of metabolic pathways (ko01100), biosynthesis of secondary metabolites (ko01110), plant hormone signal transduction (ko04075), plant-pathogen interaction (ko04626), MAPK signaling pathway (ko04010), ribosome (ko03010), biosynthesis of cofactors (ko01240), carbon metabolism (ko01200), protein processing in endoplasmic reticulum (ko04141), and phenylpropanoid biosynthesis (ko00940), among others (Supplementary Figure S1). Taken together, these analyses provided an important resource for identifying specific processes, functions, and pathways associated with heat tolerance in chickpea. 

### 2.6. Differentially Expressed Transcription Factor (TF) Families

The expression of heat stress-responsive genes was found to be regulated by specific TF gene families. A total of 800 TF encoding genes belonging to 53 different families were found to be differentially expressed (Supplementary Table S7). For instance, genes coding for bHLH (79), ERF (71), WRKY (54), MYB (51), MYB_related (40), C2H2 (38), NAC (38), bZIP (34), HD-ZIP (29), and MIKC-MADS (27), among others, were the most over-represented TF families.

### 2.7. Candidate Genes Underlying QTLs Governing Heat Tolerance in Chickpea

**Figure 4:**
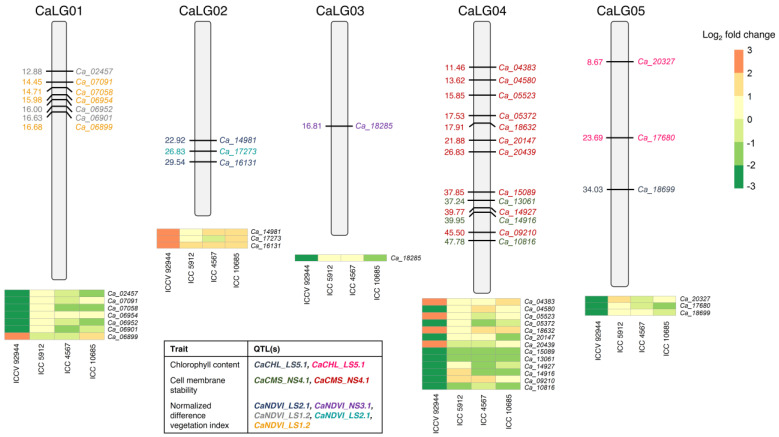
Location of prioritized candidate genes on the chickpea genome and their differential expression profiles in heat-tolerant and -sensitive genotypes. The genomic positions of the prioritized 28 genes underlying the QTLs governing heat tolerance component traits are illustrated. The colored font of the candidate genes corresponds to the QTL(s) identified for three heat tolerance component traits, as demonstrated in the table provided in the bottom-left corner of the figure. Heat maps display the differential expression of the prioritized genes in the leaves of ICCV 92944, ICC 5912, ICC 4567, and ICC 10685, before and after flowering (Supplementary Table S8). The color scale indicates log_2_-fold change.

We made an attempt to identify the candidate genes among heat-tolerant and heat-sensitive genotypes that might be responsible for regulating heat stress tolerance in chickpea. For this analysis, the QTLs (and underlying candidate genes) associated with heat tolerance component traits (e.g., chlorophyll content, cell membrane stability, and normalized difference vegetation index) reported in a previous study [7] were integrated with the differential gene expression results obtained in the present study. Since, ICCV 92944 was the heat-tolerant parent of the RIL population (DCP92-3 × ICCV 92944) that was used to detect target QTLs, we sought to identify genes that were differentially expressed in the leaves of ICCV 92944 but were not significantly regulated in sensitive genotypes (ICC 5912, ICC 4567, and ICC 10685). A total of 10 and 18 genes underlying the target QTLs were identified for leaf samples collected during the pre-anthesis and post-anthesis stages, respectively (Figure 4; Supplementary Table S8). This included genes encoding heat shock proteins (HSPs) and transcription factors (belonging to top 10 families), genes involved in photosynthesis, and those which formed an integral component of membrane. For instance, a R2R3-MYB transcription factor gene (*Ca_18699*) underlying *CaCHL_LS5.1* QTL was predicted to regulate the chlorophyll content in leaves (before flowering) in response to heat stress. A kiwifruit R2R3-MYB transcription factor, *MYB7*, was shown to play an important role in chlorophyll pigment accumulation via the transcriptional activation of metabolic pathway genes [42]. Genes associated with the normalized difference vegetation index, which represents a measure of leaf greenness (chlorophyll content), can serve as potential candidates for improving seed yield under heat stress conditions [43]. Significant differential regulation of MYB transcription factor genes (*Ca_16131* and *Ca_07058*) identified in ICCV 92944 but not in sensitive genotypes suggest the role of these genes in regulating chlorophyll content under heat stress conditions, in accordance with a previous study [44]. Furthermore, genes encoding receptor-like kinase (*Ca_13061*), major intrinsic (MIP) family transporter (*Ca_14916*), ABC transporter B (*Ca_18632*), and Leucine-Rich Repeats (LRR) receptor-like kinase (*Ca_20147*), among others, were predicted to stabilize leaf cell membrane under heat conditions. Further functional validation of these genes will help to extend our understanding of heat tolerance mechanisms in chickpea. 

## 3. Discussion

Plants, being sessile in nature, need to adapt to a range of environmental fluctuations in order to survive under short- or long-term stress conditions. A plant responds to rapid alterations in temperature conditions by qualitative and quantitative changes at the cellular and molecular levels [45]. Heat stress is among the most critical abiotic stresses that adversely affect plant development and growth, thereby causing severe yield losses. The physiological and molecular responses to heat stress have been scrutinized in agronomically important crops [46,47]. Chickpea is an important legume crop cultivated throughout the world and particularly in the dryland tropics; however, its productivity is severely influenced by heat stress [6]. In spite of the comprehensive physiological studies on high-temperature stress in chickpea, the candidate genes and molecular mechanisms associated with heat tolerance remain less explored. Understanding the molecular basis of heat stress tolerance can expedite the development of heat-tolerant chickpea varieties through genomic breeding approaches [48,49]. In the present study, we conducted a comprehensive transcriptome analysis of heat-tolerant and -sensitive genotypes at different developmental stages in order to understand the molecular response of chickpea to high-temperature stress. This study highlights the significance of scrutinizing gene expression levels across diverse genetic backgrounds and the potential of identifying key heat-responsive genes for breeding heat-tolerant chickpea varieties.

In the present study, about 97.41% of the total reads were mapped to the chickpea reference genome (CDC Frontier [14]), indicating the alignment to exonic regions. Further, the comparison of reference guided assembly with the reference genome led to the identification of 2938 (9.27% of 31,707 genes) novel genes, indicating the value of RNA-seq in the detection of novel genes. These observations confirm that the present study provided a comprehensive resource of genome-wide gene expression patterns under heat stress conditions. A hierarchical clustering of Pearson’s correlation in the transcriptome data revealed the patterns of gene transcription across different samples. Although leaf samples of all six genotypes across different stages and treatments were clustered together, root samples (after flowering) of the three tolerant genotypes under control and heat stress and ICC 4567 under heat stress formed a separate cluster. We hypothesize that this behavior is due to a specific heat stress response mechanism adopted by the heat-tolerant genotypes (and ICC 4567) after the flowering stage. Further, several hundred DEGs were identified between the tolerant and sensitive genotypes at different developmental stages under heat stress conditions. Of the 14,544 DEGs identified, a GO classification was assigned to 11,349 genes. Notably, the GO enrichment analysis provided insights into key biological processes that were associated with heat stress response in chickpea. The heat-responsive genes were predicted to be mainly involved in metabolic process, cellular process, catalytic activity, and electron transfer activity, and they were putatively localized to the cell membrane, organelle, and different cell parts. These findings are in accordance with the heat stress response observed in rice [50], wheat [51], and pearl millet [52]. Based on the KEGG pathway analysis, genes associated with heat tolerance were found to be mainly involved in metabolic pathways, the biosynthesis of secondary metabolites, and plant hormone signal transduction, similar to previous findings [51,53,54]. These annotations offer a valuable resource to identify specific biological processes, pathways, and molecular functions underlying heat stress tolerance in chickpea.

**Figure 5:**
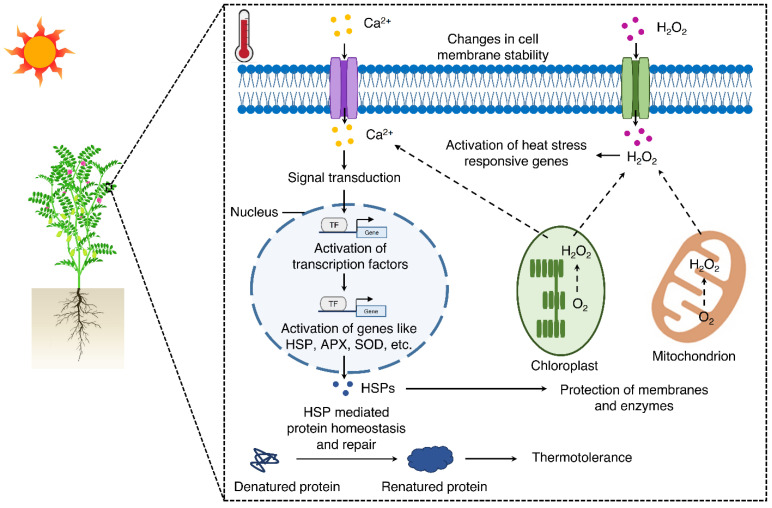
Schematic illustration of the putative regulatory networks induced in response to heat stress in chickpea. The figure summarizes the results obtained in the current study and some hypothesized mechanisms. Abbreviation for genes: HSP, heat shock protein; APX, ascorbate peroxidase; SOD, superoxide dismutase. Abbreviation for secondary messengers: Ca^2+^, calcium; H_2_O_2_, hydrogen peroxide.

Several studies have shown that calcium, which is an important secondary messenger in modulating plant growth and developmental processes, plays a major role in abiotic stress signaling [55]. In plant cells, primary heat stress sensing mainly occurs at the plasma membrane, and Ca^2+^ entry across the membrane is suggested to trigger the downstream signaling cascade [56,57]. An increase in Ca^2+^ levels in plant cells upon exposure to heat stress leads to a change in the expression levels of multiple genes, including Ca^2+^ sensors such as calmodulins (CaM) and CaM-like proteins (CMLs) along with responders such as Ca^2+^-dependent protein kinases (CDPKs) and Ca^2+^/CaM-dependent protein kinases (CCaMKs) [35]. Ca^2+^ that is majorly obtained from CaM binding kinases and phosphatases regulates the expression of HSPs by altering the activity of heat shock transcription factors [58]. In the current study, genes involved in calcium signaling pathways, including calcium-permeable stress-gated cation channel 1, calcium-binding protein, and calmodulin-like protein, showed significant differential regulation in the tolerant genotypes but not in the sensitive genotypes. These genes are predicted to activate the downstream calcium signaling pathway and impart thermotolerance in chickpea (Figure 5). An important component of plant signaling pathways is kinases, which are predicted to be ubiquitously involved in diverse signaling responses. The mitogen-activated protein kinase (MAPK) cascade is one of the major pathways operating in plants in response to heat stress through Ca^2+^ signaling [59]. A recent study showed that a tomato MAPK protein (SlMPK1) regulates antioxidants defense and functions as a negative regulator of heat stress tolerance in tomato [60]. In the present study, the MAPK signaling pathway represented one of the most enriched pathways in response to heat stress. Also, genes encoding MAPK, MAPKK, and several other kinases, including calcium/calmodulin-dependent protein kinase, LRR receptor-like serine/threonine-protein kinase, CBL-interacting protein kinase 18, etc., were found to be differentially regulated in the tolerant genotypes in response to heat stress. 

Several transcription factor genes are regulated under heat stress conditions in plants. For example, the transcription factor family genes including WRKY, MYB, AP2/ERF, bHLH, and NAC were found to be differentially regulated by heat during anthesis in rice cultivars [50]. Also, the transcription factor MYB30 was found to modulate heat stress responses in *A. thaliana* via calcium signaling [61]. In accordance with these results, in the present study, genes coding for bHLH, ERF, WRKY, MYB, C2H2, and NAC were strongly regulated under heat stress conditions. These findings suggest that genes encoding transcription factors may play an important role in the response to heat stress in tolerant chickpea genotypes (Figure 5). Furthermore, under high-temperature stress conditions, HSPs act as molecular chaperones that regulate protein folding, assembly, and degradation to minimize foreseeable damage [35]. In the current study, a large number of HSPs (including HSP 90.5, HSP 83, and small HSPs) were found to be differentially regulated in tolerant genotypes in response to high-temperature stress. These HSPs may be involved in imparting tolerance to heat stress in chickpea (Figure 5). For instance, in a previous study, overexpression of wheat chloroplastic small HSP 26 in *A. thaliana* was found to confer thermotolerance by regulating photosystem II activity, photosynthetic pigment accumulation, and increased biomass and seed yield [62]. The expression of HSPs induced in response to heat stress, are not only important for improving thermotolerance but also crucial for heat acclimation [63]. The prompt assembly of HSPs in the sensitive organelles (e.g., chloroplast and mitochondria) plays a critical role in protecting the cellular metabolic apparatus, hence acting as a major factor in the adaptation of a plant to, and survival under, high-temperature conditions [64]. In the current study, HSPs displayed a significant regulation in all six genotypes under stress conditions, suggesting a fundamental response to heat stress. 

Heat-induced transcriptional variations in the tolerant genotypes included a strong differential regulation of genes involved in metabolic processes and signal transduction pathways. The former suggests a remodeling of secondary metabolism by modulating the expression of genes such as serine carboxypeptidase in order to overcome the impacts of high temperature on the cell membrane (Figure 5). In addition, genes coding for LRR and receptor-like kinases were regulated by heat stress in the tolerant genotypes, which may account for alterations in signal transduction pathways associated with a prompt and successful heat response in chickpea. Heat stress also induced the activity of reactive oxygen species (ROS), scavenging enzymes like peroxidase, catalase and multiple antioxidants in the tolerant genotypes. Furthermore, the stress hormones ABA, brassinosteroids, and ethylene, among others, were found to be involved in signaling under high temperatures and interacted via complex regulatory networks to modulate heat stress responses in plants [65]. In the current study, genes associated with ABA and ethylene metabolism/signaling were differentially regulated under heat stress in the tolerant genotypes, but not in the sensitive genotypes. In a previous study, ABA was identified to induce heat stress tolerance in chickpea by promoting the accumulation of osmoprotectants including proline, glycine betaine, and trehalose [66]. The *abscisic acid insensitive 1 (abi1)* and *abi2* mutants of *Arabidopsis* showed a reduced survival percentage in response to high temperature conditions [67], highlighting the role of ABA in regulating heat stress response in plants. Furthermore, a previous study indicated that the *Arabidopsis* mutants *ethylene resistant 1 (etr1)* and *ethylene insensitive 2 (ein2)* were highly sensitive to high-temperature stress. In a recent study, two interacting ethylene response factors (*ERF95* and *ERF97*) were found to play an important role in regulating basal thermotolerance in *A. thaliana* [68]. Taken together, these findings pave the way for developing heat stress-tolerant chickpea varieties for the dryland tropics.

## 4. Materials and Methods

### 4.1. Plant Material, Growth Conditions, and Heat Stress Treatment Imposition

The seeds of six chickpea genotypes (three tolerant: ICCV 92944, ICC 1356, ICC 15614; and three sensitive: ICC 5912, ICC 4567, ICC 106885) were germinated under controlled conditions as described by Devasirvatham et al. [6]. The tolerance and susceptibility of the six genotypes to heat stress was defined based on various heat tolerance component traits, including pollen viability screens, heat tolerance index, time to flowering, shoot biomass, and seed yield under high temperature stress [6,10,11]. Plants of each genotype were grown in five replicates each for collecting tissues at the vegetative and reproductive stages. Three seeds of each genotype were sown in a pot (2.4 L volume) containing a mixture of black soil (vertisol):sand:vermicompost (4:2:1 by volume). Later, seedlings were thinned to one plant per pot, and the plants were grown at 28/16 °C in a glasshouse for 20 days before being transferred to a growth chamber to expose them to high temperatures at the vegetative stage. The control plants were allowed to grow in the glasshouse at 28/16 °C. The temperature in the growth chamber was increased daily by 1 °C, e.g., 28 °C to 40 °C during the day and 16 °C to 25 °C during the night, exposing the plants to a gradual increase in temperature for stress imposition. Leaf and root tissues at the vegetative stage were harvested 15 days after heat stress imposition. Similarly, for the reproductive stage, the plants were grown under glasshouse conditions until the first appearance of flowers. The plants were then subjected to heat stress in a growth chamber for 15 days, as described above. Leaf and root tissues were harvested at the reproductive stage. Tissues collected before flowering were denoted as before-flowering leaf (BFL) and before-flowering root (BFR); whereas, tissues collected after flowering were denoted as after-flowering leaf (AFL) and after-flowering root (AFR). At least three biological replicates of each tissue sample were harvested and stored at −80 °C until RNA extraction.

### 4.2. RNA Extraction, Illumina Sequencing, and Quality Check of the Sequenced Reads

Total RNA was isolated from the harvested tissues (both control and stressed samples) using TRIzol (Invitrogen; Thermo Fisher Scientific, Waltham, Massachusetts, USA) reagent according to the manufacturer’s protocol. Quality of all the samples was assessed on 1.2% formaldehyde agarose gel, while quantification was conducted by measuring A260/A280 ratio in Nanovue. Library preparation and sequencing (paired-end) was performed on an Illumina MiSeq platform following the manufacturer’s instructions. The raw reads obtained from the sequencing of all samples were subjected to quality filtering using NGS-QCbox [69] and Trimmomatic v0.35 [70]. The low-quality reads (Phred score < 20; read length < 50 bases) and reads with adapter contamination were removed in order to generate a set of high-quality reads/clean data. After stringent QC filtering, a total of 451.70 million high-quality reads were retained for subsequent analysis. 

### 4.3. Alignment of RNA-Seq Reads to the Chickpea Reference Genome

The filtered high-quality reads were mapped to the chickpea reference genome (CDC Frontier; [14]) using Tophat v2.1.0 [71], with default parameters. The mapped reads for each sample were assembled using Cufflinks v2.2.1 [72] in order to generate reference-guided assemblies. These reference-guided assemblies were then merged to generate a consensus assembly using Cuffmerge. The consensus assembly was used for all downstream analyses. 

### 4.4. Identification of DEGs, GO Enrichment and Pathway Analyses

Transcript abundance was calculated based on fragments per kilobase of transcript per million mapped reads (FPKM). Transcripts with FPKM ≥ 1 in any of the samples and a quantification status of ‘OK’ were only considered to be expressed and were considered for further analysis. Global gene expression analysis and hierarchical clustering was performed using the ‘pheatmap’ package in R software. Transcripts with FPKM ≥ 1 were log_2_ transformed, and hierarchical clustering was performed. Samples were further clustered based on their pairwise correlations. The variations in the relative abundance of the genes across different genotypes/treatments/tissues were calculated using Cuffdiff [73]. A gene was considered to be differentially expressed if it exhibited a log_2_-fold change of ≥ 2 (up-regulated) or ≤ −2 (down-regulated). In order to capture all possible DEGs, a differential expression analysis was performed using 104 different pairwise combinations. The following scheme was used for selecting 104 pairwise combinations: (i) heat stress sample/control sample from same genotype/stage/tissue (24 combinations); (ii) heat stress sample/heat stress sample from same genotype/tissue at different stage (12 combinations); (iii) each sample/sample of same stage/tissue from all other genotypes (60 combinations); (iv) control sample of tolerant genotype/control sample of sensitive genotype from same stage/tissue (8 combinations).

In order to determine the putative functions of the genes, the identified DEGs were subjected to BLASTX similarity searches (*E*-value < 1 × 10^−5^) against NCBI non-redundant protein databases, followed by annotation using Blast2GO [74]. GO enrichment analysis for DEGs was performed using the R-based GOseq package [75]. Pathway analysis of the DEGs was performed using the KEGG database [41]. To identify the transcription factor encoding genes, the DEGs were searched against the Plant transcription factor database (PlantTFDB 4.0) database with an *E*-value < 1 × 10^−5^. 

## Figures and Tables

**Table 1:** Summary of the Illumina sequencing data and read statistics of the samples.

S. No.	Sample ID	Accession	Stage	Tissue	Treat-ment	Total Reads	Filtered Reads	Filtered Reads (%)	Mapped Reads	Mapped Reads (%)
1	92944_BFL_C	ICCV 92944	Vegetative	Leaf	Control	10,535,724	10,396,767	98.68	10,200,059	98.11
2	92944_BFL_S		Vegetative	Leaf	Stress	9,748,624	9,586,491	98.34	9,420,776	98.27
3	92944_BFR_C		Vegetative	Root	Control	9,219,200	9,109,880	98.81	8,903,056	97.73
4	92944_BFR_S		Vegetative	Root	Stress	7,212,904	7,145,108	99.06	6,941,474	97.15
5	92944_AFL_C		Reproductive	Leaf	Control	12,186,436	12,019,055	98.63	11,785,230	98.05
6	92944_AFL_S		Reproductive	Leaf	Stress	10,763,618	10,651,458	98.96	10,462,620	98.23
7	92944_AFR_C		Reproductive	Root	Control	8250,522	8,127,931	98.51	7,808,259	96.07
8	92944_AFR_S		Reproductive	Root	Stress	5,524,034	5,385,805	97.50	5,014,912	93.11
9	1356_BFL_C	ICC 1356	Vegetative	Leaf	Control	13,092,636	12,902,779	98.55	12,623,893	97.84
10	1356_BFL_S		Vegetative	Leaf	Stress	7,848,062	7,762,543	98.91	7,631,234	98.31
11	1356_BFR_C		Vegetative	Root	Control	8,840,168	8,731,055	98.77	8,530,613	97.70
12	1356_BFR_S		Vegetative	Root	Stress	7,331,290	7,249,290	98.88	7,071,163	97.54
13	1356_AFL_C		Reproductive	Leaf	Control	13,595,606	13,428,551	98.77	13,186,265	98.20
14	1356_AFL_S		Reproductive	Leaf	Stress	14,108,618	13,902,500	98.54	13,657,584	98.24
15	1356_AFR_C		Reproductive	Root	Control	9,227,816	9,070,316	98.29	8,845,298	97.52
16	1356_AFR_S		Reproductive	Root	Stress	11,544,052	11,227,552	97.26	11,026,008	98.20
17	15614_BFL_C	ICC 15614	Vegetative	Leaf	Control	10,665,666	10,509,803	98.54	10,299,447	98.00
18	15614_BFL_S		Vegetative	Leaf	Stress	9,627,154	9,487,221	98.55	9,310,571	98.14
19	15614_BFR_C		Vegetative	Root	Control	9,227,608	8,942,970	96.92	8,672,250	96.97
20	15614_BFR_S		Vegetative	Root	Stress	9,642,178	9,537,244	98.91	9,229,981	96.78
21	15614_AFL_C		Reproductive	Leaf	Control	8,068,362	7,934,679	98.34	7,776,680	98.01
22	15614_AFL_S		Reproductive	Leaf	Stress	7,523,960	7,432,553	98.79	7,246,601	97.50
23	15614_AFR_C		Reproductive	Root	Control	4,552,154	4,432,821	97.38	4,332,522	97.74
24	15614_AFR_S		Reproductive	Root	Stress	8,145,728	8,044,853	98.76	7,394,860	91.92
25	5912_BFL_C	ICC 5912	Vegetative	Leaf	Control	7,969,184	7,869,032	98.74	7,618,997	96.82
26	5912_BFL_S		Vegetative	Leaf	Stress	7,941,190	7,856,097	98.93	7,716,168	98.22
27	5912_BFR_C		Vegetative	Root	Control	10,438,074	10,085,541	96.62	9,785,839	97.03
28	5912_BFR_S		Vegetative	Root	Stress	8,414,738	8,336,666	99.07	8,141,952	97.66
29	5912_AFL_C		Reproductive	Leaf	Control	10,500,550	10,363,949	98.70	10,182,627	98.25
30	5912_AFL_S		Reproductive	Leaf	Stress	5,180,758	5,117,767	98.78	4,961,472	96.95
31	5912_AFR_C		Reproductive	Root	Control	6,801,290	6,728,029	98.92	6,557,444	97.46
32	5912_AFR_S		Reproductive	Root	Stress	11,591,808	11,410,935	98.44	11,130,213	97.54
33	4567_BFL_C	ICC4567	Vegetative	Leaf	Control	12,433,794	12,216,153	98.25	11,994,301	98.18
34	4567_BFL_S		Vegetative	Leaf	Stress	10,425,446	10,288,128	98.68	10,068,543	97.87
35	4567_BFR_C		Vegetative	Root	Control	9,393,346	9,167,255	97.59	8,885,865	96.93
36	4567_BFR_S		Vegetative	Root	Stress	5,773,594	5,725,855	99.17	5,541,685	96.78
37	4567_AFL_C		Reproductive	Leaf	Control	12,591,206	12,398,886	98.47	12,166,369	98.12
38	4567_AFL_S		Reproductive	Leaf	Stress	9,909,176	9,778,957	98.69	9,596,564	98.13
39	4567_AFR_C		Reproductive	Root	Control	13,244,230	13,065,481	98.65	12,701,915	97.22
40	4567_AFR_S		Reproductive	Root	Stress	7,681,168	7,497,257	97.61	7,175,938	95.71
41	10685_BFL_C	ICC 10685	Vegetative	Leaf	Control	10,826,998	10,674,415	98.59	10,455,869	97.95
42	10685_BFL_S		Vegetative	Leaf	Stress	11,806,122	11,612,804	98.36	11,332,255	97.58
43	10685_BFR_C		Vegetative	Root	Control	11,425,388	11,082,467	97.00	10,756,797	97.06
44	10685_BFR_S		Vegetative	Root	Stress	7,308,052	7,241,501	99.09	7,058,819	97.48
45	10685_AFL_C		Reproductive	Leaf	Control	12,762,970	12,546,086	98.30	12,296,546	98.01
46	10685_AFL_S		Reproductive	Leaf	Stress	10,978,522	10,853,672	98.86	10,661,986	98.23
47	10685_AFR_C		Reproductive	Root	Control	7,721,326	7,641,066	98.96	7,464,657	97.69
48	10685_AFR_S		Reproductive	Root	Stress	9,251,526	9,124,015	98.62	8,884,704	97.38
	Total					458,852,576	451,701,239	98.45	440,508,881	97.41

**Table 2:** Genes that exhibit significant differential expression in the tolerant genotypes but are not significantly regulated in the sensitive genotypes.

Gene ID	XLOC ID	Log_2_ Fold Change (Treatment/Control)						Gene Description
		ICCV 92944	ICC 1356	ICC 15614	ICC 5912	ICC 4567	ICC 10685	
Leaf sample—before flowering								
*Ca_14776*	XLOC_001145	−2.161	−2.165	−2.125	−1.702	−1.651	1.532	pathogenesis-related protein 10
*Ca_07845*	XLOC_009218	−2.956	2.458	−2.134	−0.806	1.402	−0.163	adenylate isopentenyltransferase
Leaf sample—after flowering								
*Ca_00611*	XLOC_000503	3.140	−3.321	2.981	−0.057	0.576	1.887	basic 7S globulin
*Ca_00716, Ca_00717*	XLOC_007162	−2.600	2.662	2.696	0.615	1.725	−0.349	IST1-like protein isoform X2
*Ca_05434*	XLOC_010012	−2.650	2.103	4.128	−0.786	1.774	−0.593	protein trichome birefringence-like 36
*Ca_10969*	XLOC_012559	−2.618	2.066	2.539	1.526	1.894	0.050	selenoprotein H
*Ca_11344*	XLOC_014356	−2.875	2.951	2.332	0.728	1.912	NA	nuclear transcription factor Y subunit C-4
*Ca_25834*	XLOC_017871	3.984	2.594	2.935	0.960	0.517	1.430	glycine-rich cell wall structural 1-like
*Ca_05233*	XLOC_019232	−2.424	2.341	2.077	1.273	0.213	−0.392	beta-xylosidase/alpha-L-arabinofuranosidase 2
*Ca_06616*	XLOC_022893	−2.089	2.078	2.323	0.228	1.379	−0.698	DUF936 family protein
*Ca_01970*	XLOC_025377	−2.259	2.122	2.621	1.089	1.683	0.103	chalcone-flavanone isomerase family
*Ca_27831*	XLOC_030374	−2.519	2.197	2.352	0.991	1.566	1.025	CTP synthase-like isoform X1
*Ca_00464*	XLOC_000429	2.041	−3.878	−3.219	0.674	NA	−0.680	serine carboxypeptidase-like 11
*Ca_06899*	XLOC_002705	2.490	−2.172	−2.465	−0.001	−0.193	1.441	NAC family transcription factor 4
*Ca_09600*	XLOC_018907	2.256	−2.235	−2.951	−0.953	−0.489	−0.075	transcription factor TGA4-like isoform X1
*Ca_02987*	XLOC_021039	2.420	−9.719	−4.680	0.607	−0.407	1.176	ABA-responsive protein ABR18-like
Root sample—before flowering								
*Ca_14147*	XLOC_000968	4.027	2.467	2.073	1.012	NA	1.305	cysteine-rich repeat secretory protein 38-like
*Ca_03546*	XLOC_009482	3.704	3.129	2.302	1.785	1.538	0.734	peroxidase 5-like
*Ca_06976, Ca_06977*	XLOC_001062	−2.850	−2.721	−2.895	0.538	0.289	−0.728	UDP-glycosyltransferase 79B30
*Ca_06902*	XLOC_002703	−3.674	2.910	−2.009	0.407	−1.651	1.136	PREDICTED: uncharacterized protein LOC101506036
*Ca_14738*	XLOC_002759	−2.465	−2.426	−2.305	−1.309	−1.193	NA	E3 ubiquitin- ligase LIN-1
*Ca_19160*	XLOC_003847	2.254	−2.392	−3.952	−1.107	−0.690	−1.771	arabinogalactan peptide 21-like
*Ca_18792*	XLOC_006264	−2.067	−2.650	−3.088	−0.820	−1.456	−0.846	dirigent protein 9-like
*Ca_16612*	XLOC_010291	−2.866	−2.265	−3.618	−1.585	−1.241	−0.737	probable pectate lyase 1
*Ca_23747*	XLOC_013214	−2.464	−2.961	−2.093	−1.327	NA	0.074	probable pectinesterase/pectinesterase inhibitor 25
*Ca_22983*	XLOC_026437	−2.194	3.352	−2.720	1.481	−1.969	1.948	hypothetical protein glysoja_025159
Root sample—after flowering								
*Ca_11978*	XLOC_007090	−2.016	−2.800	2.140	−0.071	−0.706	−0.682	ras-related protein RABE1c-like
*Ca_18112*	XLOC_003876	2.567	2.437	−2.526	1.302	−1.692	1.597	mitochondrial uncoupling protein 5
*Ca_09783*	XLOC_005932	2.229	−2.034	−2.205	−0.125	NA	−0.189	OBERON-like protein
*Ca_05504*	XLOC_009968	−2.783	−2.225	−2.072	−0.018	−0.801	−0.283	glutamine--tRNA ligase
*Ca_24258*	XLOC_022284	−3.033	2.178	−3.424	0.380	NA	0.127	NA
*NA*, not available								

## Data Availability

The sequence data have been deposited in the Sequence Read Archive (SRA) database at NCBI under the accession number PRJNA748749.

## Supplementary Materials

The following supporting information can be downloaded at: https://www.mdpi.com/article/10.3390/ijms24021369/s1.
