# Supplementary material for: Comprehensive Transcriptome Profiling Uncovers Molecular Mechanisms and Potential Candidate Genes Associated with Heat Stress Response in Chickpea
Source: Int J Mol Sci. 2023 Jan 10;24(2):1369. doi: 10.3390/ijms24021369 (PMC9865869; doi:10.3390/ijms24021369)
Supplement: Supplementary file 1 [file ijms-24-01369-s001.zip › Supplementary Figure S1.pdf]

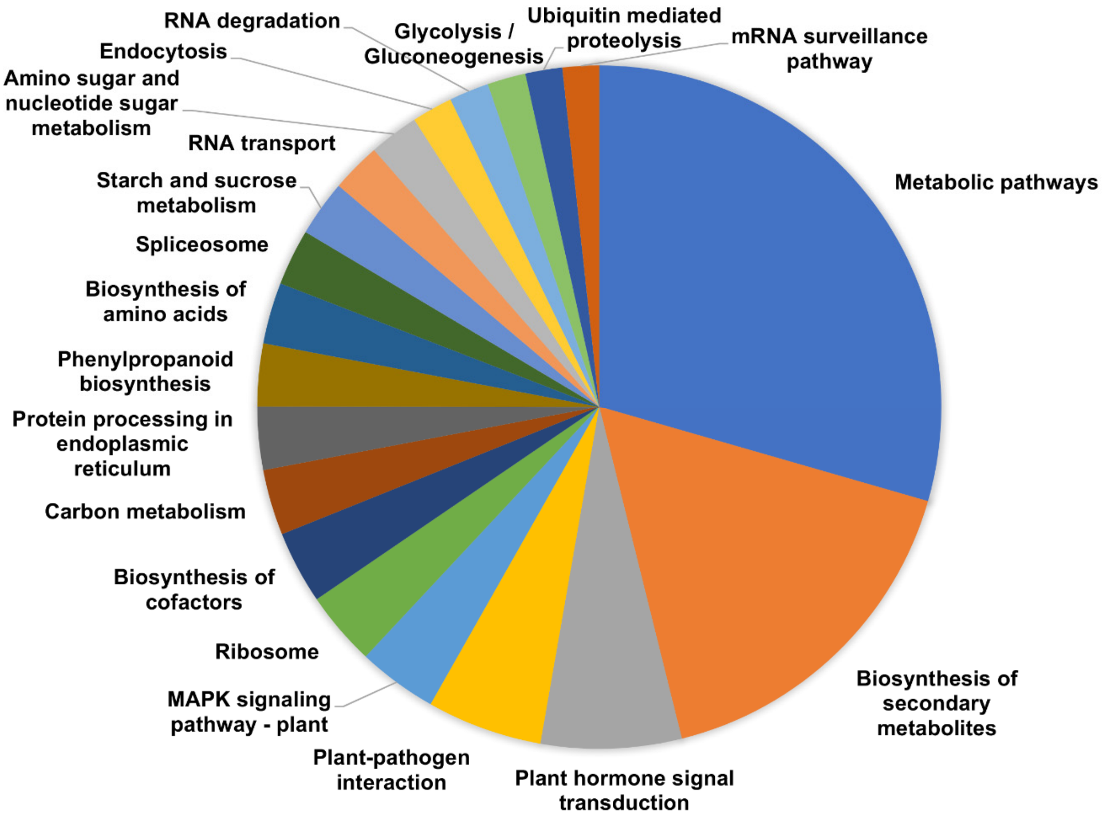

Supplementary Figure S1: KEGG pathway analysis of differentially expressed genes (DEGs) under heat stress in chickpea. The pie chart represents major pathways represented by DEGs under high temperature stress conditions in chickpea.
